# Mapping EQ5D utilities from forced vital capacity and diffusing capacity in fibrotic interstitial lung disease

**DOI:** 10.1371/journal.pone.0283110

**Published:** 2023-03-31

**Authors:** Alyson W. Wong, Huiying Sun, Ingrid A. Cox, Jolene H. Fisher, Nasreen Khalil, Kerri A. Johannson, Veronica Marcoux, Deborah Assayag, Helene Manganas, Martin Kolb, Andrew J. Palmer, Barbara de Graaff, E. Haydn Walters, Peter Hopkins, Christopher Zappala, Nicole S. Goh, Yuben Moodley, Vidya Navaratnam, Tamera J. Corte, Christopher J. Ryerson, Wei Zhang

**Affiliations:** 1 Department of Medicine, University of British Columbia, Vancouver, British Columbia, Canada; 2 Centre for Heart Lung Innovation, St. Paul’s Hospital, Vancouver, British Columbia, Canada; 3 Centre for Health Evaluation and Outcome Sciences, Vancouver, British Columbia, Canada; 4 Menzies Institute for Medical Research, University of Tasmania, Hobart, Tasmania, Australia; 5 Department of Medicine, University of Toronto, Toronto, ON, Canada; 6 Department of Medicine, University of Calgary, Calgary, AB, Canada; 7 Department of Medicine, University of Saskatchewan, Saskatoon, Saskatchewan, Canada; 8 Department of Medicine, McGill University, Montreal, Quebec, Canada; 9 Département de Médecine, Centre Hospitalier de l’Université de Montréal, Montreal, QC, Canada; 10 Department of Medicine, Firestone Institute for Respiratory Health, McMaster University, Hamilton, ON, Canada; 11 School of Medicine, University of Tasmania, Hobart, Tasmania, Australia; 12 Lung Transplant Unit, The Prince Charles Hospital, Brisbane, Australia; 13 Dept of Thoracic Medicine, Royal Brisbane and Women’s Hospital, Brisbane, Australia; 14 Dept of Respiratory and Sleep Medicine, Austin Hospital, Heidelberg, Australia; 15 Department of Respiratory Medicine, Fiona Stanley Hospital, Murdoch, Australia; 16 Department of Respiratory and Sleep Medicine, Royal Prince Alfred Hospital, Sydney, NSW, Australia; 17 Centre of Research Excellence for Pulmonary Fibrosis, University of Sydney, Sydney, NSW, Australia; 18 School of Population and Public Health, University of British Columbia, Vancouver, British Columbia, Canada; SMS Medical College and Hospital, INDIA

## Abstract

**Objectives:**

Fibrotic interstitial lung disease (ILD) includes a large group of conditions that lead to scarring of the lungs. The lack of available 5-level EuroQol 5D (EQ5D) data has limited the ability to conduct economic evaluations in ILD. The purpose of this study was to develop and validate a mapping algorithm that predicts EQ5D utilities from commonly collected pulmonary function measurements (forced vital capacity [FVC] and diffusing capacity of the lung for carbon monoxide [DLCO]) in fibrotic ILDs.

**Methods:**

EQ5D utility and pulmonary function measurements from the Canadian Registry for Pulmonary Fibrosis were included. Ordinary least squares (OLS), beta regression, two-part, and tobit models were used to map EQ5D utilities from FVC or DLCO. Model performance was assessed by comparing the predicted and observed utilities. Subgroup analyses were also conducted to test how well models performed across different patient characteristics. The models were then externally validated in the Australian Idiopathic Pulmonary Fibrosis Registry.

**Results:**

The OLS model performed as well as other more complex models (root mean squared error: 0.17 for FVC and 0.16 for DLCO). As with the other models, the OLS algorithm performed well across the different subgroups (except for EQ5D utilities < 0.5) and in the external validation cohort.

**Conclusion:**

We developed a mapping algorithm that predicts EQ5D utilities from FVC and DLCO, with the intent that this algorithm can be applied to clinical trial populations and real-world cohorts that have not prioritized collection of health-related utilities. The mapping algorithm can be used in future economic evaluations of potential ILD therapies.

## Introduction

Fibrotic interstitial lung disease (ILD) is an umbrella term used for a large group of diseases that cause scarring (fibrosis) of the lungs. These diseases typically result in decline in lung function, worsening quality of life, and early mortality [1]. The economic burden of ILD is significant. The annual direct medical costs for patients with idiopathic pulmonary fibrosis (IPF), a common type of fibrotic ILD, is two-fold higher than age- and sex-matched healthy controls [2]. Recent studies show that antifibrotic therapies, which were initially indicated in IPF only, also slow lung function decline among different progressive fibrotic ILDs [3, 4]. This expanded indication for antifibrotic therapy will significantly increase the costs associated with ILD, and it is therefore critical to determine whether drugs or other health interventions are cost-effective.

Quality-adjusted life-years (QALYs) are one of the most common outcome measures in cost-effectiveness analyses. In order to calculate QALYs, a health-related quality of life (QoL) measure such as the EuroQol 5D (EQ5D) is required. The EQ5D contains 5 QoL dimensions: mobility, self-care, usual activities, pain/discomfort, and anxiety/depression. For each of these dimensions, patients grade the severity of problems based on five levels: no, slight, moderate, severe, and extreme problems [5]. A value set is then used to convert the EQ5D rating to a health utility that is used to calculate a QALY [6]. A value set is a scoring algorithm derived from a country-specific validation study that anchors a health utility of 1 as “perfect health” and 0 as “death” [7]. Thus, the range of possible health utilities differ between countries based on these value sets. For example, if a person rated “no problems” in all domains, their EQ5D rating would be “11111.” Using the country specific value set for the normal population, the Canadian health utility would be 0.949, while the Australian health utility would be 1.

The EQ5D is an important measure as some organizations responsible for evaluating drugs and health technologies prefer its use in economic evaluations [8]; however, EQ5D utility data in ILD are extremely limited because they are not routinely collected. The lack of available EQ5D utilities has been a major limiting factor in conducting cost-effectiveness analyses in ILD. When utilities are unavailable, “mapping” offers a solution to determine utilities from other measures of health outcomes through the development of models or algorithms. The percent predicted forced vital capacity (FVC) and diffusing capacity for carbon monoxide (DLCO) are pulmonary function measurements that are routinely collected to assess disease severity and progression in ILD. We sought to develop and validate a mapping algorithm that could predict EQ5D utilities from FVC or DLCO in fibrotic ILDs. The ability to generate EQ5D utilities from FVC and DLCO data allows the comparison of health impacts of different interventions in terms of quality-adjusted life years (QALYs), which is a commonly used effectiveness measure in cost-effectiveness analyses. The results of cost-effectiveness analyses are crucial to making informed funding decisions.

## Methods

### Study populations

The Canadian Registry for Pulmonary Fibrosis (CARE-PF) was used to develop the mapping algorithm. This prospective cohort includes patients over 18 years of age with any fibrotic ILD who can provide informed consent and complete study questionnaires in English or French. There are no exclusion criteria. Patients are recruited from eight Canadian ILD centres and seen in follow-up as clinically indicated (typically every 3–6 months) with standardized questionnaires collected at each visit [9]. Patients with idiopathic pulmonary fibrosis (IPF), hypersensitivity pneumonitis (HP), connective tissue disease-associated ILD (CTD-ILD), and unclassifiable ILD were included in this analysis.

External validation of the mapping algorithms was conducted using the Australian Idiopathic Pulmonary Fibrosis Registry (AIPFR), a multi-center prospective observational cohort of patients with IPF [10]. Those who had EQ5D and PFT data were included in the validation cohort. Ethics approval for this project was obtained and participants provided written informed consent (UBC #H19-01989).

### Measurements

Baseline patient characteristics were collected from patient-completed questionnaires. Patients enrolled in CARE-PF complete the EQ5D survey at each clinic visit. The EQ5D score was converted into a health utility using a Canadian value set [6]. All pulmonary function tests and EQ5D results that were within 3 months of one another were included. If a patient had more than one PFT-EQ5D pairing within 3 months, then the pair with the shortest time interval was used. Thus, a single patient could have multiple PFT-EQ5D pairings collected over time and each of these discrete pairs were used in the analysis and treated independently. The AIPFR collected the EQ5D once from patients who consented to complete the questionnaire. Using the Global Lung Function Initiative prediction equations, the FVC and DLCO were mapped to utilities as they are routinely collected pulmonary function measurements and predictive of survival in ILD [11–14].

### Model development and internal validation

A total of 5 models were derived using the following statistical techniques: ordinary least squares (OLS), beta regression, two-part model with OLS, two-part model with beta regression, and tobit. These models were chosen based on those used in previous mapping studies and the distribution of the EQ5D utilities in the two analyzed cohorts [15–17]. Models also included commonly collected patient data, including age, sex, smoking pack-years, and ILD subtype.

Ten-fold cross-validation was used to internally validate each model in the CARE-PF cohort. The original sample was randomly partitioned into 10 groups, and the model was derived using 9 groups and tested in the remaining test group. This process was repeated 10 times such that each observation was included in the test group once. Model fit was assessed using the root mean squared error (RMSE) and mean absolute error (MAE), which compare the observed and predicted utilities. The RMSE and MAE were averaged across the test groups, with values closer to 0 representing better model fit. Model performance was also explored among subgroups based on stratifications by age, sex, ILD subtype, EQ5D utilities, FVC and DLCO.

### External validation

The mapping algorithms from all models were applied to the AIPFR cohort and the predicted EQ5D utilities were compared to the observed using the RMSE and MAE. The AIPFR EQ5D responses were converted to utilities using both the Australian and Canadian value set to explore how different value sets impacted model performance [6, 18].

### Recommended model

The recommended model was determined based on discussion amongst a multidisciplinary team comprised of a health economist, statistician, and ILD clinicians. Selection of the final mapping algorithm considered model performance in the original and external cohorts, as well as performance across subgroups. The RMSE and MAE were compared to other mapping studies which have typically reported values < 0.2 [15–17]. The ease of understanding and applying the model were also factored into the decision. All analyses were conducted using R statistical software (version 4.0.3).

## Results

### Patient characteristics

**Fig 1:**
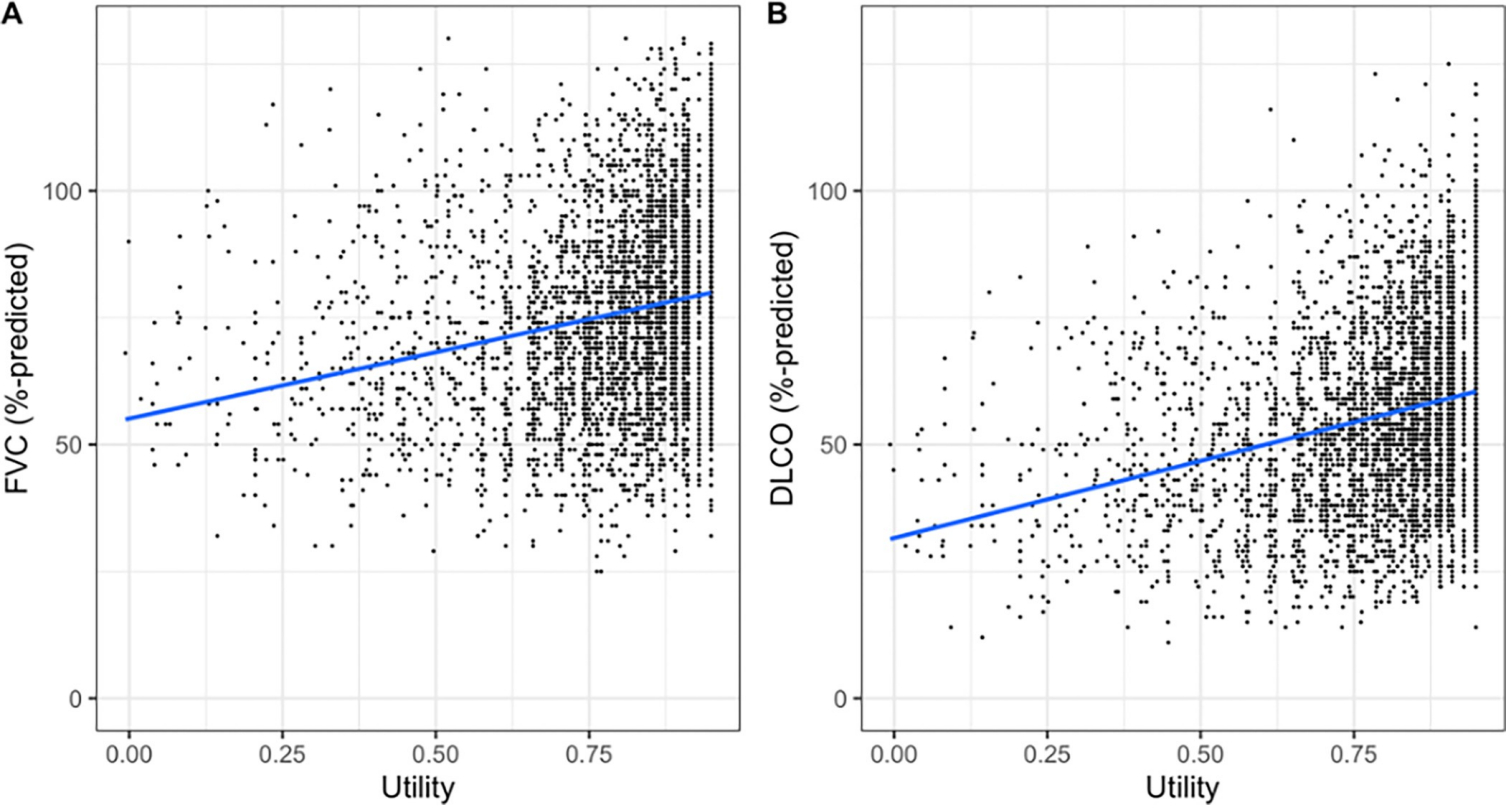
Relationship between FVC or DLCO and Canadian EQ5D utilities in CARE-PF.

The CARE-PF cohort consisted of 2,307 patients (Table 1). There were 5,325 EQ5D-FVC observations and 4,345 EQ5D-DLCO observations, with no meaningful differences between patients who provided data for FVC and DLCO analysis (S1 Table). The cohort was comprised of 51% males with a mean age of 66 ± 12 years. The most common ILD subtypes were CTD-ILD (40%) and IPF (32%). Patients had mild-moderate disease at baseline based on the FVC and DLCO. The median baseline EQ5D utility was 0.8 (IQR 0.7–0.9) with the ability to perform usual activities being the most impacted quality of life domain (i.e., the highest proportion of people reported at least slight impairment in this domain). The relationships of FVC and DLCO with EQ5D utilities in the CARE-PF cohort are shown in Fig 1. The AIPFR cohort had a higher proportion of males, was older in age, and had a lower median EQ5D utility compared to CARE-PF.

**Table 1:** Baseline patient characteristics.

Characteristics	CARE-PF	AIPFR
	n = 2,307	n = 81
Age, years	65 ± 12	74 ± 7
Male	1168 (51)	52 (64)
Smoking pack-years	5 (0–27)	3 (0–20)
ILD type, n (%)		
IPF	743 (32)	81 (100)
CTD-ILD	910 (40)	-
HP	215 (9)	-
Unclassifiable	439 (19)	-
Lung function		
FVC, %-predicted	75 ± 20	88 ± 22
DLCO, %-predicted	57 ± 19	49 ± 16
EQ5D score ≥ 2, n (%)		
Mobility	1,303 (56)	46 (57)
Self-care	638 (28)	17 (21)
Usual activities	1,441 (62)	53 (65)
Pain or discomfort	1,287 (56)	53 (65)
Anxiety or depression	1,125 (49)	30 (37)
EQ5D VAS	70 (55–84)	70 (60–85)
EQ5D utility	0.8 (0.7–0.9)	0.7 (0.6–0.9)

Values represent mean ± standard deviation, number (percent), or median (interquartile range). An EQ5D score ≥ 2 represents at least a slight problem reported in the EQ5D domain. Abbreviations: BMI, body mass index; FVC, forced vital capacity; DLCO, diffusing capacity of the lung for carbon monoxide; IPF, idiopathic pulmonary fibrosis; CTD-ILD, connective tissue disease-associated ILD; HP, hypersensitivity pneumonitis.

### Model development and internal validation

The beta regression model had the lowest RMSE and MAE; however, overall, the average RMSE and MAE for the 5 models were similar when mapping from FVC or DLCO to EQ5D utilities (Table 2). The range of predicted utilities was the widest for the beta regression and tobit models. The models also performed similarly for FVC and DLCO among subgroups based on age, sex, ILD subtypes, and disease severity, with the average RMSE and MAE < 0.21 (S2 and S3 Tables). The only subgroup in which the models did not perform as well in were patients with EQ5D utilities ≤ 0.5 with the average RMSE and MAE being approximately 0.4.

**Table 2:** Observed versus predicted utilities when mapping FVC or DLCO to Canadian EQ5D utilities using the CARE-PF cohort.

			Min	Mean	Max	RMSE	MAE
**FVC** n = 2,304	**Observed utility**		**-0.1038**	**0.7797**	**0.9489**	**-**	**-**
	**Predicted utilities**	OLS	0.6011	0.7798	0.9745	0.1709	0.1270
		Beta	0.5619	0.7855	0.8957	0.1703	0.1249
		Two-part OLS	0.6014	0.7797	0.9356	0.1707	0.1267
		Two-part Beta	0.6012	0.7773	0.9014	0.1708	0.1276
		Tobit	0.5815	0.7719	0.9064	0.1706	0.1287
**DLCO** n = 1,938	**Observed utility**		**-0.0064**	**0.7928**	**0.9489**	**-**	**-**
	**Predicted utilities**	OLS	0.6380	0.7928	0.9698	0.1606	0.1184
		Beta	0.6262	0.7982	0.8957	0.1602	0.1168
		Two-part OLS	0.6323	0.7928	0.9359	0.1604	0.1181
		Two-part Beta	0.6012	0.7773	0.9014	0.1605	0.1189
		Tobit	0.6227	0.7855	0.9087	0.1603	0.1199

### External validation

The lowest RMSE and MAE were seen with the tobit model at 0.23 and 0.17, respectively, when mapping from FVC to Australian EQ5D utilities, and 0.22 and 0.16 when mapping from DLCO. However, the RMSE and MAE for the simpler OLS model (0.24 and 0.18 for FVC; 0.22 and 0.16 for DLCO) were similar to the tobit model. In general, the range of predicted utilities was narrower in the AIPFR cohort than in the CARE-PF cohort.

We explored how the models performed when different value sets were used to calculate the observed utilities among patients with IPF in CARE-PF or AIPR cohorts (Table 3; S4 and S5 Tables). These observed utilities were then compared to the predicted utilities to assess model fit. The RMSE and MAE values were lowest when the Canadian value set was applied to the AIPFR cohort (approximate RMSE 0.12 and MAE 0.08 for FVC and DLCO). This was followed by the CARE-PF cohort using the Canadian value set (approximate RMSE 0.11–0.15 and MAE 0.10–0.14 for FVC and DLCO). The highest RMSE and MAE occurred when the Australian value set was applied to AIPFR (approximate RMSE 0.23 and MAE 0.17 for FVC and DLCO).

**Table 3:** Comparison of model performance when mapping FVC or DLCO to EQ5D utilities using different value sets in the CARE-PF and AIPFR cohorts.

		CARE-PF using Canadian value set		AIPFR using Canadian value set		AIPFR using Australian value set	
		RMSE	MAE	RMSE	MAE	RMSE	MAE
**FVC** n = 2,304	**OLS**	0.1559	0.1425	0.1274	0.0870	0.2387	0.1796
	**Beta**	0.1551	0.1425	0.1211	0.0834	0.2310	0.1754
	**Two-part OLS**	0.1557	0.1422	0.1240	0.0847	0.2352	0.1777
	**Two-part Beta**	0.1573	0.1441	0.1219	0.0851	0.2294	0.1737
	**Tobit**	0.1557	0.1425	0.1219	0.0849	0.2291	0.1736
**DLCO** n = 1,938	**OLS**	0.1164	0.1047	0.1219	0.0861	0.2239	0.1647
	**Beta**	0.1158	0.1058	0.1211	0.0848	0.2235	0.1649
	**Two-part OLS**	0.1160	0.1044	0.1211	0.0849	0.2239	0.1647
	**Two-part Beta**	0.1224	0.1114	0.1215	0.0861	0.2229	0.1649
	**Tobit**	0.1182	0.1071	0.1221	0.0880	0.2207	0.1625

### Recommended models

Overall, OLS performed as well as the other models when using FVC or DLCO values to predict EQ5D utilities and is easier to understand and apply from a clinical perspective. OLS also performed similarly in subgroup analyses and when applied to the external AIPFR cohort. Thus, the recommended OLS model was applied to the entire CARE-PF cohort and resulted in the mapping algorithms below. All variables are continuous except for male sex and ILD subtypes (CTD-ILD, HP, and Unclassifiable) which are categorical, with IPF as the reference ILD subtype. Examples of how to apply the mapping algorithms are provided in the S1 File.

For use of FVC to predict EQ5D utility:

EQ5D utility = 0.5986 + 0.0026*FVC + 0.0001*Age + 0.0213*Male– 0.0012*Smoking pack-years– 0.0170*CTD-ILD– 0.0068*HP– 0.0219*Unclassifiable

For use of DLCO to predict EQ5D utility:

EQ5D utility = 0.5599 + 0.0028*DLCO + 0.0016*Age + 0.0082*Male– 0.0007*Smoking pack-years– 0.0249*CTD-ILD– 0.0215*HP– 0.0380*Unclassifiable

## Discussion

To our knowledge, this is the first algorithm that maps FVC or DLCO to EQ5D utilities in ILD. We used a large, national, prospective ILD registry that included eight sites across Canada. With the large number of EQ5D and FVC/DLCO observations, we were able to divide the cohort into training and test cohorts for internal validation, and subsequently externally validated findings using a large cohort from a second country. In addition, we demonstrated that the model overall performed well in various subgroups. Although direct collection of health-related utility data is preferred over the use of mapping algorithms, such utility data are infrequently available. Our algorithm is therefore an important tool using commonly available measurements to enable economic evaluation that would otherwise not be able to be conducted.

The purpose of this study was to identify a mapping algorithm that performed well and could be employed in health economics and clinical research. In order to demonstrate generalizability, we showed that the OLS model performed well in a variety of clinical subgroups that were stratified by age, sex, and ILD subtype. We also considered fit across the range of plausible utility values, rather than just around the mean utility. The OLS model fit well to the mean and upper range of the observed utilities; but, like the other models we tested, the OLS model did not perform as well when the observed EQ5D utility was ≤ 0.5. Ideally, the model would fit well across the full range of utilities; however, published studies and clinical trials tend to enrol patients with mild to moderate disease. A mapping algorithm that performs well for patients with severe disease and lower EQ5D utilities is therefore less relevant as most data for economic evaluation of medical devices or therapies are based on clinical trials. Furthermore, the health utilities for patients with ILD (even those with end-stage disease) typically fall within the range of utilities where the OLS model performed well. In a cost-effectiveness analysis of antifibrotic therapy in IPF, the utilities for a patient whose disease was stable, progressive, or end-stage and requiring lung transplant were 0.847, 0.782, and 0.7, respectively [19].

A strength of our study was the ability to externally validate the model in an Australian IPF cohort and explore how predicted utilities compared to observed utilities using different value sets (i.e., weights). The range of observed utilities based on different value sets was as follows: AIPFR/Canadian weight (0.2153–0.9489), CARE-PF/Canadian weight (0.038–0.9489), and AIPFR/Australian weight (-0.1740–1). Interestingly, as with the other models, the OLS model performed best when the Canadian weight was applied to AIPFR. This is most likely due to the model not performing as well with lower observed EQ5D utilities and the lower end of the EQ5D utilities being the highest for the AIPFR/Canadian weight combination. This highlights the importance of exploring how mapping algorithms perform using different value sets. Although the OLS model performed best with the AIPFR/Canadian weight combination, it did well with the other combinations (RMSE and MAE < 0.2) and its performance was comparable to other mapping studies.

This study has several limitations. First, the Australian cohort comprised patients with IPF only. The OLS model performed well among the different ILD subtypes in the CARE-PF cohort, but external validation for these ILD subtypes is required. Second, assessing the mapping algorithm’s performance in economic evaluation is required. This could be achieved by comparing the outcomes of a cost effectiveness study using primary health utility data versus the predicted utilities. Third, it is possible that another model structure that was not included in this study may fit better. Mapping studies have typically evaluated a few model structures, with OLS being the most common [20]. In addition to OLS, we also included beta regression, two-part, and tobit models, which allowed us to identify the best algorithm after exploring a diverse range of model structures that have been successful in past mapping studies and were appropriate for the distribution of the utilities. The RMSE and MAE values for the final recommended OLS model were also comparable to other mapping studies [15–17]. Lastly, there was variability around the line of best fit seen in the correlation plots between FVC or DLCO and EQ5D (Fig 1), suggesting a variable relationship between these PFT measurements and EQ5D at an individual level. However, the purpose of this mapping algorithm is to support economic evaluation using large populations, making the variability at the individual level less critical than the model’s overall performance at a population level.

## Conclusion

Our study provides the first mapping algorithm for FVC or DLCO measurements to EQ5D utilities in patients with fibrotic ILD. The recommended OLS algorithm demonstrated good performance among different patient subgroups and was externally validated in patients with IPF. This robust algorithm that connects pulmonary function measurements to EQ5D can be applied to clinical trial populations and real-world cohorts that have not prioritized collection of health-related utilities and is critical to supporting economic evaluations of potential ILD therapies.

## Supporting information

S1 Table Patient characteristics of CARE-PF cohort based on those used to derive the FVC or DLCO model. (DOCX)

S2 Table Comparison of FVC models applied to subgroups within CARE-PF using the Canadian value set. Subgroups included different severities of quality of life (EQ5D), ILD subtypes, sex, age, and ILD severity based on lung function (FVC). Abbreviations: FVC, forced vital capacity; IPF, idiopathic pulmonary fibrosis; CTD-ILD, connective tissue disease-associated ILD; HP, hypersensitivity pneumonitis.
(DOCX)

S3 Table Comparison of DLCO models applied to subgroups within CARE-PF using the Canadian value set. Subgroups included different severities of quality of life (EQ5D), ILD subtypes, sex, age, and ILD severity based on lung function (DLCO). Abbreviations: DLCO, diffusing capacity of the lung for carbon monoxide; IPF, idiopathic pulmonary fibrosis; CTD-ILD, connective tissue disease-associated ILD; HP, hypersensitivity pneumonitis.
(DOCX)

S4 Table Observed utilities compared to predicted utilities when mapping FVC or DLCO to EQ5D utilities using the Australian value set in the AIPFR cohort. (DOCX)

S5 Table Observed utilities compared to predicted utilities when mapping FVC or DLCO to EQ5D utilities using the Canadian value set in the AIPFR cohort. (DOCX)

S1 File Example of how to apply the mapping algorithms. (DOCX)
