# Supplementary material for: Mapping EQ5D utilities from forced vital capacity and diffusing capacity in fibrotic interstitial lung disease
Source: PLoS One. 2023 Mar 31;18(3):e0283110. doi: 10.1371/journal.pone.0283110 (PMC10065299; doi:10.1371/journal.pone.0283110)
Supplement: S1 Table — (DOCX) [file pone.0283110.s001.docx]

**Table S1. Patient characteristics of CARE-PF cohort based on those used to derive the FVC or DLCO model.**

| **Characteristics** | **CARE-PF**  **FVC subgroup**  **n=2,304** | **CARE-PF**  **DLCO subgroup**  **n=1,938** |
| --- | --- | --- |
| Age, years | 66 ± 12 | 65 ± 12 |
| Male | 1166 (51) | 979 (51) |
| Smoking pack-years | 5 (0-27) | 5 (0-27) |
| ILD type, n (%)  IPF  CTD-ILD  HP  Unclassifiable | 742 (32)  908 (39)  215 (9)  439 (19) | 584 (30)  789 (41)  182 (9)  383 (20) |
| Lung function  FVC, %-predicted  DLCO, %-predicted | 75 ± 20  57 ± 19 | 77 ± 19  57 ± 19 |
| EQ5D score ≥ 2, n (%)  Mobility  Self-care  Usual activities  Pain or discomfort  Anxiety or depression | 1,301 (56)  637 (28)  1,439 (62)  1,286 (56)  1,123 (49) | 1047 (54)  492 (25)  1163 (60)  1071 (55)  920 (47) |
| EQ5D VAS | 70 (55-84) | 70 (60-85) |
| EQ5D utility | 0.8 (0.7-0.9) | 0.8 (0.7-0.9) |
