# Supplementary material for: Mapping EQ5D utilities from forced vital capacity and diffusing capacity in fibrotic interstitial lung disease
Source: PLoS One. 2023 Mar 31;18(3):e0283110. doi: 10.1371/journal.pone.0283110 (PMC10065299; doi:10.1371/journal.pone.0283110)
Supplement: S2 Table — Subgroups included different severities of quality of life (EQ5D), ILD subtypes, sex, age, and ILD severity based on lung function (FVC). Abbreviations: FVC, forced vital capacity; IPF, idiopathic pulmonary fibrosis; CTD-ILD, connective tissue disease-associated ILD; HP, hypersensitivity pneumonitis. (DOCX) [file pone.0283110.s002.docx]

**Table S2. Comparison of FVC models applied to subgroups within CARE-PF using the Canadian value set.** Subgroups included different severities of quality of life (EQ5D), ILD subtypes, sex, age, and ILD severity based on lung function (FVC). Abbreviations: FVC, forced vital capacity; IPF, idiopathic pulmonary fibrosis; CTD-ILD, connective tissue disease-associated ILD; HP, hypersensitivity pneumonitis.

| **Subgroup** | **RMSE** | | | | | **MAE** | | | | |
| --- | --- | --- | --- | --- | --- | --- | --- | --- | --- | --- |
|  | **OLS** | **Beta** | **Two-part OLS** | **Two-part Beta** | **Tobit** | **OLS** | **Beta** | **Two-part OLS** | **Two-part Beta** | **Tobit** |
| **EQ5D < 0.5** | 0.4264 | 0.4344 | 0.4267 | 0.4120 | 0.4204 | 0.4043 | 0.4122 | 0.4045 | 0.3894 | 0.3979 |
| **EQ5D 0.5-0.75** | 0.1342 | 0.1388 | 0.1342 | 0.1193 | 0.1281 | 0.1090 | 0.1157 | 0.1093 | 0.0950 | 0.1037 |
| **EQ5D > 0.75** | 0.1129 | 0.1060 | 0.1124 | 0.1265 | 0.1176 | 0.0961 | 0.0901 | 0.0957 | 0.1105 | 0.1011 |
| **IPF** | 0.1559 | 0.1551 | 0.1557 | 0.1573 | 0.1557 | 0.1164 | 0.1158 | 0.1160 | 0.1224 | 0.1182 |
| **CTD** | 0.1706 | 0.1701 | 0.1705 | 0.1715 | 0.1704 | 0.1274 | 0.1255 | 0.1271 | 0.1330 | 0.1291 |
| **HP** | 0.1725 | 0.1727 | 0.1725 | 0.1736 | 0.1726 | 0.1306 | 0.1290 | 0.1305 | 0.1354 | 0.1322 |
| **Unclassifiable** | 0.1939 | 0.1939 | 0.1937 | 0.1940 | 0.1935 | 0.1417 | 0.1367 | 0.1418 | 0.1461 | 0.1432 |
| **Male** | 0.1685 | 0.1683 | 0.1682 | 0.1694 | 0.1683 | 0.1243 | 0.1226 | 0.1241 | 0.1301 | 0.1263 |
| **Female** | 0.1731 | 0.1724 | 0.1729 | 0.1739 | 0.1728 | 0.1292 | 0.1269 | 0.1289 | 0.1343 | 0.1306 |
| **Age < 65** | 0.1747 | 0.1748 | 0.1746 | 0.1759 | 0.1747 | 0.1299 | 0.1282 | 0.1296 | 0.1356 | 0.1317 |
| **Age >= 65** | 0.1678 | 0.1670 | 0.1676 | 0.1685 | 0.1675 | 0.1245 | 0.1223 | 0.1243 | 0.1298 | 0.1261 |
| **FVC < 50** | 0.2081 | 0.2088 | 0.2081 | 0.2065 | 0.2070 | 0.1608 | 0.1600 | 0.1608 | 0.1617 | 0.1611 |
| **FVC 50 - 80** | 0.1782 | 0.1780 | 0.1782 | 0.1798 | 0.1786 | 0.1360 | 0.1329 | 0.1357 | 0.1410 | 0.1374 |
| **FVC > 80** | 0.1440 | 0.1426 | 0.1435 | 0.1446 | 0.1431 | 0.1018 | 0.1012 | 0.1016 | 0.1094 | 0.1044 |
