# Supplementary material for: Mapping EQ5D utilities from forced vital capacity and diffusing capacity in fibrotic interstitial lung disease
Source: PLoS One. 2023 Mar 31;18(3):e0283110. doi: 10.1371/journal.pone.0283110 (PMC10065299; doi:10.1371/journal.pone.0283110)
Supplement: S3 Table — Subgroups included different severities of quality of life (EQ5D), ILD subtypes, sex, age, and ILD severity based on lung function (DLCO). Abbreviations: DLCO, diffusing capacity of the lung for carbon monoxide; IPF, idiopathic pulmonary fibrosis; CTD-ILD, connective tissue disease-associated ILD; HP, hypersensitivity pneumonitis. (DOCX) [file pone.0283110.s003.docx]

**Table S3. Comparison of DLCO models applied to subgroups within CARE-PF using the Canadian value set.** Subgroups included different severities of quality of life (EQ5D), ILD subtypes, sex, age, and ILD severity based on lung function (DLCO). Abbreviations: DLCO, diffusing capacity of the lung for carbon monoxide; IPF, idiopathic pulmonary fibrosis; CTD-ILD, connective tissue disease-associated ILD; HP, hypersensitivity pneumonitis.

| **Subgroups** | **RMSE** | | | | | **MAE** | | | | |
| --- | --- | --- | --- | --- | --- | --- | --- | --- | --- | --- |
|  | **OLS** | **Beta** | **Two-part OLS** | **Two-part Beta** | **Tobit** | **OLS** | **Beta** | **Two-part OLS** | **Two-part Beta** | **Tobit** |
| **EQ5D < 0.5** | 0.4272 | 0.4365 | 0.4267 | 0.4130 | 0.4212 | 0.4084 | 0.4182 | 0.4078 | 0.3937 | 0.4021 |
| **EQ5D 0.5-0.75** | 0.1376 | 0.1435 | 0.1379 | 0.1239 | 0.1324 | 0.1156 | 0.1231 | 0.1159 | 0.1022 | 0.1107 |
| **EQ5D > 0.75** | 0.1049 | 0.0978 | 0.1045 | 0.1169 | 0.1088 | 0.0880 | 0.0827 | 0.0876 | 0.1007 | 0.0923 |
| **IPF** | 0.1425 | 0.1425 | 0.1422 | 0.1441 | 0.1425 | 0.1047 | 0.1058 | 0.1044 | 0.1114 | 0.1071 |
| **CTD** | 0.1641 | 0.1637 | 0.1640 | 0.1648 | 0.1639 | 0.1211 | 0.1193 | 0.1209 | 0.1261 | 0.1226 |
| **HP** | 0.1663 | 0.1662 | 0.1662 | 0.1670 | 0.1661 | 0.1233 | 0.1205 | 0.1233 | 0.1279 | 0.1244 |
| **Unclassifiable** | 0.1740 | 0.1739 | 0.1736 | 0.1742 | 0.1734 | 0.1291 | 0.1252 | 0.1289 | 0.1327 | 0.1300 |
| **Male** | 0.1579 | 0.1577 | 0.1576 | 0.1587 | 0.1576 | 0.1170 | 0.1156 | 0.1168 | 0.1221 | 0.1186 |
| **Female** | 0.1630 | 0.1628 | 0.1629 | 0.1639 | 0.1628 | 0.1193 | 0.1178 | 0.1192 | 0.1247 | 0.1209 |
| **Age < 65** | 0.1680 | 0.1681 | 0.1678 | 0.1688 | 0.1678 | 0.1240 | 0.1219 | 0.1238 | 0.1289 | 0.1253 |
| **Age >= 65** | 0.1541 | 0.1536 | 0.1538 | 0.1549 | 0.1538 | 0.1135 | 0.1125 | 0.1132 | 0.1189 | 0.1152 |
| **DLCO < 50** | 0.1862 | 0.1865 | 0.1861 | 0.1863 | 0.1859 | 0.1435 | 0.1408 | 0.1435 | 0.1462 | 0.1443 |
| **DLCO 50 - 80** | 0.1459 | 0.1457 | 0.1459 | 0.1479 | 0.1462 | 0.1069 | 0.1055 | 0.1066 | 0.1136 | 0.1089 |
| **DLCO > 80** | 0.1170 | 0.1136 | 0.1153 | 0.1141 | 0.1135 | 0.0763 | 0.0793 | 0.0757 | 0.0835 | 0.0788 |
