# Supplementary material for: Mapping EQ5D utilities from forced vital capacity and diffusing capacity in fibrotic interstitial lung disease
Source: PLoS One. 2023 Mar 31;18(3):e0283110. doi: 10.1371/journal.pone.0283110 (PMC10065299; doi:10.1371/journal.pone.0283110)
Supplement: S5 Table — (DOCX) [file pone.0283110.s005.docx]

**Table S5. Observed utilities compared to predicted utilities when mapping FVC or DLCO to EQ5D utilities using the Canadian value set in the AIPFR cohort.**

|  |  |  | **Min** | **Mean** | **Max** | **RMSE** | **MAE** |
| --- | --- | --- | --- | --- | --- | --- | --- |
| **FVC**  n=2,304 | **Observed utility** | | **0.2153** | **0.8213** | **0.9489** | **-** | **-** |
|  | **Predicted utilities** | OLS | 0.7017 | 0.8392 | 1.0358 | 0.1261 | 0.0867 |
|  |  | Beta | 0.7055 | 0.8276 | 0.9122 | 0.1198 | 0.0832 |
|  |  | Two-part OLS | 0.7023 | 0.8355 | 0.9570 | 0.1227 | 0.0844 |
|  |  | Two-part Beta | 0.7110 | 0.8218 | 0.9211 | 0.1206 | 0.0849 |
|  |  | Tobit | 0.6952 | 0.8218 | 0.9267 | 0.1207 | 0.0847 |
| **DLCO**  n=1,938 | **Observed utility** | | **0.2153** | **0.8224** | **0.9489** | **-** | **-** |
|  | **Predicted utilities** | OLS | 0.7029 | 0.8100 | 0.9399 | 0.1207 | 0.0858 |
|  |  | Beta | 0.7072 | 0.8080 | 0.8835 | 0.1198 | 0.0845 |
|  |  | Two-part OLS | 0.7020 | 0.8111 | 0.9221 | 0.1199 | 0.0846 |
|  |  | Two-part Beta | 0.7123 | 0.8060 | 0.8901 | 0.1204 | 0.0858 |
|  |  | Tobit | 0.6943 | 0.8011 | 0.8959 | 0.1209 | 0.0877 |
