# Supplementary material for: Mapping EQ5D utilities from forced vital capacity and diffusing capacity in fibrotic interstitial lung disease
Source: PLoS One. 2023 Mar 31;18(3):e0283110. doi: 10.1371/journal.pone.0283110 (PMC10065299; doi:10.1371/journal.pone.0283110)
Supplement: S1 File — (DOCX) [file pone.0283110.s006.docx]

**Example of how to apply the mapping algorithms:**

For use of FVC to predict EQ5D utility:

EQ5D utility = 0.5986 + 0.0026*FVC + 0.0001*Age + 0.0213*Male – 0.0012*Smoking pack-years – 0.0170*CTD-associated ILD – 0.0068*HP – 0.0219*Unclassifiable

Example: A 67 year old male with IPF and 25 pack-years smoking history with a FVC of 75%-predicted would have a predicted EQ5D utility of 0.79 (0.5986 + 0.0026*75+0.0001*67 + 0.0213*1 – 0.0012*25).

For use of DLCO to predict EQ5D utility:

EQ5D utility = 0.5599 + 0.0028*DLCO + 0.0016*Age + 0.0082*Male – 0.0007*Smoking pack-years – 0.0249*CTD-associated ILD – 0.0215*HP – 0.0380*Unclassifiable

Example: A 50 year old female with a CTD-ILD and 10 pack-years smoking history with a DLCO of 60%-predicted would have a predicted EQ5D utility of 0.78 (0.5599 + 0.0028*60+ 0.0016*50 + 0.0082*0 – 0.0007*10 – 0.0249*1).
